# User experience of a family health history chatbot: A quantitative analysis

**DOI:** 10.21203/rs.3.rs-2886804/v1

**Published:** 2023-05-03

**Authors:** Caitlin Allen

**Keywords:** Family Health History, Health Information Technology, Chatbot, User Experience, Telemetry, User Behavior

## Abstract

**Objective:**

Family health history (FHx) is an important tool in assessing one’s risk towards specific health conditions. However, user experience of FHx collection tools is rarely studied. ItRunsInMyFamily.com (ItRuns) was developed to assess FHx and hereditary cancer risk. This study reports a quantitative user experience analysis of ItRuns.

**Methods:**

We conducted a public health campaign in November 2019 to promote FHx collection using ItRuns. We used software telemetry to quantify abandonment and time spent on ItRuns to identify user behaviors and potential areas of improvement.

**Results:**

Of 11065 users who started the ItRuns assessment, 4305 (38.91%) reached the final step to receive recommendations about hereditary cancer risk. Highest abandonment rates were during Introduction (32.82%), Invite Friends (29.03%), and Family Cancer History (12.03%) subflows. Median time to complete the assessment was 636 seconds. Users spent the highest median time on Proband Cancer History (124.00 seconds) and Family Cancer History (119.00 seconds) subflows. Search list questions took the longest to complete (median 19.50 seconds), followed by free text email input (15.00 seconds).

**Conclusion:**

Knowledge of objective user behaviors at a large scale and factors impacting optimal user experience will help enhance the ItRuns workflow and improve future FHx collection.

## 1. Introduction

Family health history (FHx) is an important tool for assessing a patient’s risks of specific health conditions such as cancer. Knowledge of FHx could help healthcare professionals determine the potential risks for an individual and recommend targeted intervention and preventive measures. Web-based, patient-facing tools have been developed to systematically collect and organize FHx outside of the provider’s office, improving efficiency of clinical visits while increasing completeness and accuracy.^1–4^ These tools allow users to generate pedigree, collaborate with family, provide decision support, and support health information technology (HIT) integration.

Automated conversational agents (chatbots) have emerged as a promising approach for health data collection. Chatbots promote a friendly, intuitive, and engaging user experience.^5,6^ One such effort is ItRunsInMyFamily.com (ItRuns) - a web-based, simple, and scalable tool that engages users in a conversation using a chat interface to collect FHx and provide recommendations regarding hereditary cancer risks.^3^ Research has demonstrated data collection chatbots (such as ItRuns) reduce the workload on users and are perceived as more usable and satisfactory compared to standard web-based forms, even when collecting granular FHx.^6^

Collection of FHx can involve detailed questions and significant time investment, making it imperative to provide users with an optimal and engaging experience in using FHx tools. Very few studies have explored the user experience of these tools.^1^ Research has found that FHx collection tools can produce poor user satisfaction with respect to assessment format, data-input method, and complex clinical jargon.^2^ Patients’ incomplete knowledge of family members’ health history and confidentiality concerns remain as barriers to FHx tool adoption.^7,8^ In addition, factors such as time taken to collect comprehensive FHx and types and amount of data inputs could also lead to under-utilization and poor user experience.

Research has focused on understanding user experience and preferences employing qualitative and experimental approaches such as surveys, interviews, comparative assessments, and randomized control trials.^2–4, 6,9^ The purpose of this study is to explore quantitative software telemetry (collection of software usage information) to assess granular, objective user behaviors, abandonment (where and when a user leaves the FHx tool) trends, and time to complete various steps to identify potential pitfalls and areas of user experience improvement.^10^ To the best of our knowledge, no studies have explored user behaviors of completing FHx assessments using telemetry data. In this paper, we explore the software telemetry to assess user behaviors at a large scale and identify potential factors impacting user experience. The insights gathered from this quantitative evaluation will allow us to enhance the overall user experience of ItRuns assessment.

## 2. Methods

### 2.1 ItRunsInMyFamily.com and November 2019 10K Campaign

ItRuns is a web based FHx collection and hereditary cancer risk assessment tool developed by researchers at the Medical University of South Carolina and ItRunsInMyFamily.com LLC. ItRuns is a free, secure, browser-based, and mobile-first application that does not require the user to download software or to create an account.^3^ ItRuns uses a dialogue-based text chat interface to mimic human-to-human conversation providing its users a natural and engaging experience analogous to talking with providers. The current version of ItRuns uses an innovative chatbot interface that simulates a natural conversational dialogue to collect FHx from users. The chatbot engages users in a structured and intuitive way, rather than multiple pages of online forms, tables, or a series of complex questions compared to other tools. Upon completion of the ItRuns assessments, a personalized risk assessment report (section 3.2.1) is sent to the user’s email address.^3^

This paper reports on data collected from an online FHx collection campaign conducted using ItRuns.^11^ This campaign was conducted between November 1, 2019 and November 30, 2019 in conjunction with Family Health History Month.^11^ The ItRuns team aimed to recruit at least 10,000 English-speaking users via paid and unpaid marketing campaigns through social media promotion, contacting previous users of ItRuns, using promotional banners on the ItRuns website, and direct contact with cancer support groups. Interested users completed the assessment using the ItRuns assessment link on mobile devices or computers. Users consented to take the ItRuns assessment by clicking on consent checkboxes within the assessment. This secondary analysis study was deemed not human subject research by the Medical University of South Carolina Institutional Review Board (Pro00094990).

### 2.2 ItRunsInMyFamily Workflow

**Figure 1:**
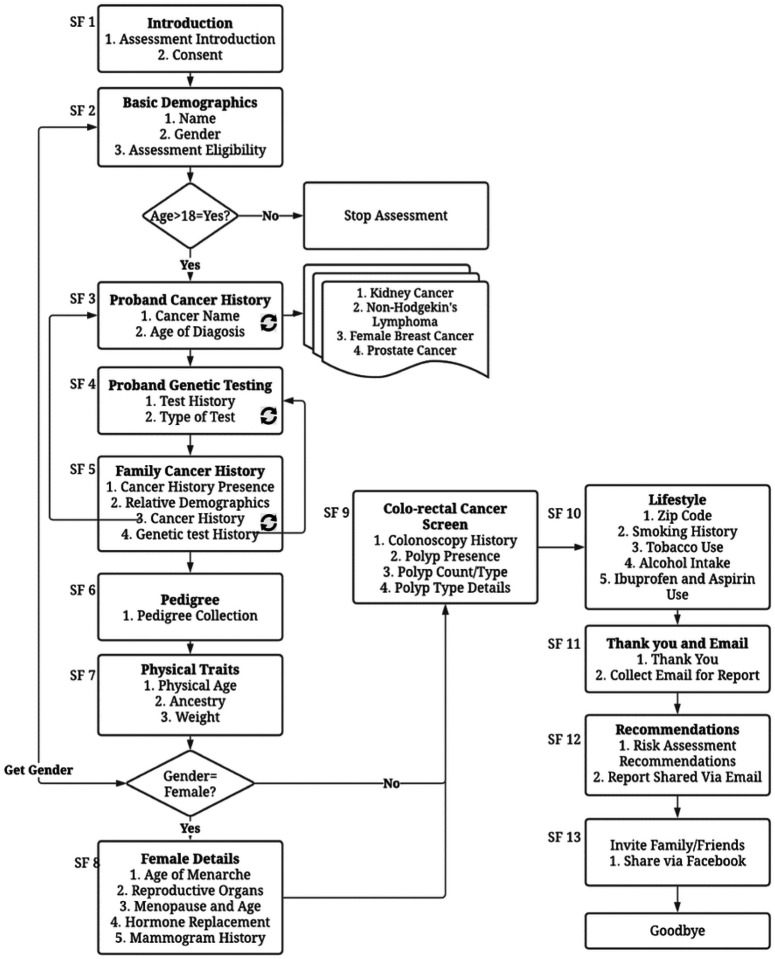
ItRuns assessment workflow

This section provides a step-by-step description of the ItRuns assessment workflow divided into 13 subflows (SF) as explained below (See Fig. 1).

SF 1 | Introduction: The assessment begins with an introduction to Dokbot - the chatbot persona users engage with during the assessment.^12^ Dokbot introduces the user to the purpose of ItRuns and obtains informed consent.
SF 2 | Basic Demographics: Upon consent, Dokbot collects the user’s name and gender. Dokbot also asks if the user is 18 years or older. If yes, Dokbot continues with the assessment. If not, the assessment ends with a message explaining that users have to be at least 18 years of age to take the assessment.
SF 3 | Patient Cancer History: This step collects users’ cancer history. If the user reports a history, Dokbot collects information related to the type of cancer and age of diagnosis. Some cancers include specific follow up questions. For example: for breast cancer, questions such as if the cancer was in one or both breasts.
SF 4 | Genetic Testing: Following the personal cancer questions, Dokbot asks the user if they have ever been tested for a genetic mutation in a cancer-causing gene. If answered yes, Dokbot asks if the result was positive and, if so, for which gene.
SF 5 | Family Cancer History: Dokbot then asks if anyone in the family has had cancer. If the user reports yes, Dokbot asks which relative, their name, cancer, age of diagnosis, as well as cancer-specific follow up questions, if the relative had any other cancers, if they are still alive, their current age or age when they died, and whether the family member has ever been tested for a genetic mutation in a cancer-causing gene. This sequence is repeated for each relative the user reports as having been diagnosed with cancer.
SF 6 | Pedigree: Dokbot asks the total number of daughters, sons, sisters, brothers, maternal aunts, maternal uncles, paternal aunts, paternal uncles in their family. Numbers of nieces and nephews were not assessed due to the complexity of the data and its limited utility. For female users, age when they had their first child is assessed during this sequence. In this manner, a 3-generation pedigree is collected.
SF 7 | Physical Traits: Dokbot collects users’ age, weight, and ancestral origin. If users indicate that they are of European descent, Dokbot asks whether they have Ashkenazi Jewish ancestry as these individuals are at a higher risk of BRCA gene mutation.^13^
SF 8 | Female Details: If the user is a female (indicated in SF 2), they are asked the age at menarche; if they have had their breasts, ovaries, or uterus removed, if they have gone through menopause and at what age; if they take hormone replacement therapy if they had a mammogram. If they have had a mammogram, Dokbot asks the date of the last mammogram and the history of dense breasts. They are also asked if they’ve had a breast biopsy, if yes, number of biopsies, any abnormal findings and type of abnormality.
SF 9 | Colorectal Cancer Screen: Dokbot asks all users about the history of colonoscopy. If yes, Dokbot further asks if polyps were found and, if so, the number and type.
SF 10 | Lifestyle: Dokbot collects a user’s zip code, smoking history, tobacco use and alcohol use. If the user reports having smoked cigarettes, Dokbot asks for their age when they started smoking and the age when they stopped; if they report they are no longer a current smoker, what number of cigarettes per day when they did smoke. Dokbot also collects ibuprofen and aspirin use.
SF 11 | Thank you and Email: Dokbot thanks the user for participation and collects the user’s email to send the ItRuns risk analysis report.
SF 12 | Recommendations and Report: Dokbot providers high-level risk recommendation based on family health history and guidelines for hereditary cancer risks. Dokbot also informs the user that a detailed report (section 2.4.1) will be shared via email.
SF 13 | Invite Friends: Users have an option to share ItRuns assessment with friends and relatives on Facebook by posting on their public profile.

#### 2.2.1 ItRuns Risk Assessment Report

ItRuns uses ontologies, ontological reasoners, clinical practice guidelines, and web services to provide evidence-based recommendations to users based on their FHx. Owlready2 ontology module and Protégé ontology platform were used to develop a lightweight, patient-centric clinical practice guideline domain ontology using hereditary cancer criteria from the American College of Medical Genetics and Genomics and the National Cancer Comprehensive Network. The development of this ontology-driven clinical practice guideline criteria risk assessment is published in a separate publication.^14^ A risk analysis PDF report is emailed to participants who completed the ItRuns assessment. The report contains an Executive Summary, followed by a Guidelines section consisting of the hereditary cancer predisposition criteria the user meets and the published recommendations for cancer predisposition assessment they should follow. Next, the Health History section includes information about the user and their health history (including a family pedigree), a breakdown of relatives with cancer, relatives who are also at risk, and family cancer statistics for the user’s family. Finally, the Recommendations section includes additional details about the hereditary cancer syndrome the user for which the user might be at risk, available genetic counseling resources, and additional genetic testing information. The About section includes content about the product, contact information, support, references, and a legal disclaimer.

### 2.3 Study Participants

Potential users were invited via paid and unpaid campaigns as described in Section 2.2. They provided consent to participate in the study at the beginning of the assessment. Adult users (≥18 years) were allowed to continue with the assessment after providing consent. Users were asked for their email address to send their Cancer Risk Assessment report at the end of the assessment. No other personal identifying information was collected.

### 2.4 Telemetry Data

During the assessment, the Dokbot software collected and stored in a relational database telemetry data detailing the user’s behavior interacting with the software, including timestamps of all actions, transitions between steps, geographical location, referral URL, browser information, and supported languages.

### 2.5 Data Analysis

Once the campaign was completed, telemetry data was extracted from the Dokbot database. A script was run to remove small amounts of unusable data, including records where a user loaded the ItRuns workflow but did not submit any single step as well as researcher/developer test records. Then, we extracted elements such as unique random user ID, step description, step start and end timestamps, etc. to assess the experience.

Using an analysis script, we quantified the telemetry data to calculate the usage, abandonment rate, and time per step. We tabulated and graphically represented this data using Microsoft Excel software. Descriptive measures were used to obtain the frequencies, percentages, mean, and median related to completion, abandonment rate, and time taken for each subflow and related steps.

## 3. Results

Between the testing period, 14,140 users clicked on our marketing campaign and landed on the ItRuns homepage. Here, we report on usage behaviors of 11,065 (78.25%) users who started the ItRuns assessment. A total of 4,305 (38.91%) users completed the assessment and reached the final step to receive a recommendation. About 575,000 Dokbot steps were presented. On average, highest departures or assessment abandonments were in the Introduction (32.82%), Invite Friends (29.03%), and Family Cancer History (12.03%) subflows. Most (89.20%) individuals left the assessment without inviting family members or friends to take the ItRuns assessment. Table 1 summarizes the key steps in the assessment workflow including departures per step, percentage abandonment, and time spent to complete the steps.

### 3.1 Overall abandonment by steps

**Figure 2:**
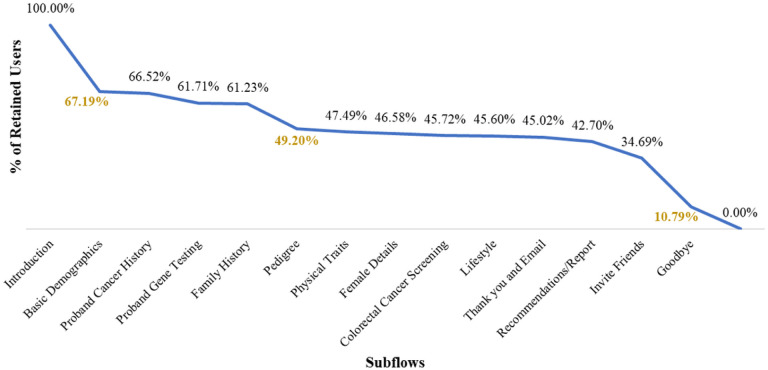
Percentage of retained users by subflows

High abandonments were seen in the Introduction subflow (32.81%) and in Invite Friends (23.90%) subflow where users were asked to invite friends and family members to complete the ItRuns assessment via Facebook. Collecting Family Cancer History subflow had the third largest drop of 12.03%. See Fig. 2.

**Figure 3:**
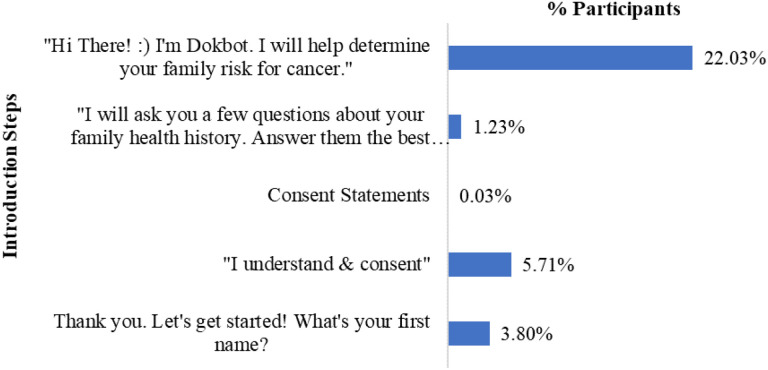
Stepwise abandonment in the Introduction subflow

All users (11,065; 100.0%) first experienced the Introduction subflow with the highest abandonment rate (32.81%). Within this subflow, 22.03% of users left at the first introduction step (Fig. 3), followed by a 5.71% drop when users were asked to agree to the consent statements of the assessment.

A high drop off from 11,065 to 3,841 showed 34.7% of all users continued to Invite Friends. However, of the 3,841 there were 2,645 (68.9%) who left the step. Therefore, 1,196 out of 11,065 (10.8%) completed Invite Friends subflow.

**Figure 4:**
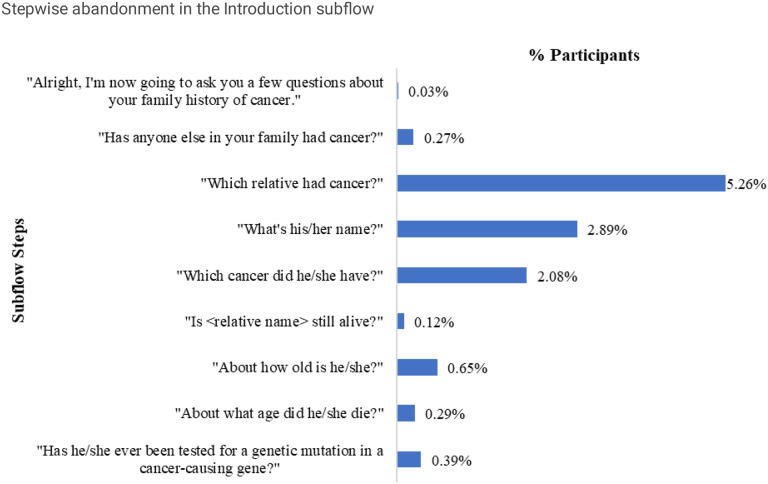
Stepwise abandonment in the Family Cancer History subflow

The third largest overall drop was seen at the Family Cancer History subflow (Fig. 4). Users may have to enter cancer history details of one or more family members (e.g., mother, father, etc.), so the user may complete all or some of the steps in this subflow multiple times. In this study, we did not determine when the user abandoned the assessment if they completed the subflow multiple times. The highest (5.26%, 43.73% of the subflow) abandonment was seen during the step when users were asked about which of their family members had cancer followed by relative’s names (2.89%) and types of cancer they had (2.08%).

### 3.2 Abandonment by steps

We looked at abandonment by individual steps as opposed to overall abandonment to identify questions that are particularly challenging to users, despite only being seen by a small percentage of users. Table 2 provides details of the top steps of abandonment.

The median time to complete the assessment was 636 seconds or 10.60 minutes. Table 1 demonstrates the total median time spent on each subflow. Users spent the highest median time to complete the Proband Cancer History (124.00 seconds) followed by Family Cancer History (119.00 seconds), Female Details (67.00), Pedigree (55.00 seconds), and Lifestyle (53.00 seconds). Users spent the least amount of time in Basic Demographics (7.00 seconds), Lifestyle (8.00 seconds), and Invite Friends (9.00 seconds) subflows.

Table 3 shows the distribution of key steps with the highest amount of time spent. Users spent the longest median time (36.00 seconds) on the question about which cancers their relatives had. They spent 20 seconds on the question asking where their ancestors were from. This checklist question included a 9-option list of geographic areas with examples (such as Americas [Central or South America, Native American]). Users spent about 17 seconds at the step calculating their risk of cancer. This step calculated the user’s risk of cancer based on guidelines and no input was required. Users also spent about 16 seconds in completing the consent step followed by 14.50 seconds in responding to the question about if their genetic test had a positive result.

### 3.4 Question types

The assessment consisted of 94 steps requiring user interaction and response. A user may or may not have interacted with 100% of the steps. Common response types are button responses, text or numerical inputs, and search lists to add information related to cancers and genetic tests. In general, Yes/No button responses were the most commonly used response type and the fastest (median time- 3.68 seconds) for users to answer with the smallest abandonment rate (0.08%). Interestingly, a variation of the Yes/No response type with an additional “I don’t know” response option had one of the highest response times (14.50 seconds) and abandonment rate of 0.28%. This response option was only used on one question, so it is unclear if this is due to the response type or the question (“Was the [*relative genetic testing*] result positive?”). Text input fields had the highest rate of drop off (3.35%) although the email input question took 15 seconds to complete. Search lists where users were required to look up details on cancer types (over 100 options) and genetic test types (160 options) took the longest to complete with a median time of 19.50 seconds. See Table 4 for more details.

## 4. Discussion

We sought to understand the user experience of ItRuns to enhance FHx collection. This paper quantitatively explores software telemetry data to identify actual user behaviors and actions using software telemetry. Our findings suggest that factors such as lack of knowledge or interest in FHx, trust in FHx tools, data sharing and confidentiality concerns about sharing personal and family health information, lack of collaboration with family members, and increased time spent on conversational text-based workflow can contribute to suboptimal user experience. We discuss potential considerations for improving user experience of FHx tools and implications for ItRuns.

### 4.1 Main findings

About one in three users (32.81%) abandoned the assessment in the initial Introduction subflow with a 22.03% drop after the greeting statement. We hypothesize that potential reasons for this drop could be the lack of knowledge or interest in cancer risk assessment and distrust or privacy concerns in using a casual, informal, chat-based agent to share personal and family health information. In a previous study, users expressed concerns regarding confidentiality of sharing FHx using digital tools.^7^ In healthcare chatbot applications, lower reading level and informal language is sometimes perceived to be less trustworthy.^15^ We observed abandonment when asked to provide their consent to begin assessment. While ItRuns ensures users that their data is protected with compliance regulations (including HIPAA, GDPR, and CCPA), concerns over sharing personal health information with a third-party solution (not directly recommended by their provider or as a part of a marketing campaign) could restrict users from moving forward. Research shows that while patients are comfortable with sharing personal health information with their own providers, they often restrict sharing information outside of their care network.^16^

Over 12% of user drop was seen in the Family Cancer History subflow. Commonly, users abandoned the assessment when asked about which relatives had cancer, their names, and types of cancer they had. As seen in previous research, users may have abandoned at this stage of the assessment if they did not have the correct or complete information about family cancer history.^4,17^ As ItRuns currently does not allow live collaboration with family members or return to complete assessment at a later time, users could abandon the assessment considering the potential lack of specific family health information. Also, the distrust of the technology and skepticism to share personal health information of family members with ItRuns (a third-party solution) could also play a role. In addition, the looping nature of this workflow required users to add one or more family members’ information one-at-a-time and with one or more occurrences of adding cancer and genetic testing information. About 7,000 users completed some steps of these workflows over 22,000 times (average of three repetitions per user). This repetitive nature of flow took an average of 119 seconds to complete with the top steps averaging 53.00 seconds.

A high (23.90%) drop was seen at the end of the workflow when users were asked if they would like to share ItRuns assessment with family members and friends via Facebook. While waiting for their risk assessment results, the assessment asked, “While you are waiting on your report, will you invite your friends and family to use ItRunsInMyFamily so they can learn their family cancer risk as well?”. Research suggests that users may not be interested in sharing health information online but rather personally via direct communication.^18^ People tend to be cautious of sharing health information online, as few formal policies exist to ensure protection of users’ information and the ease of which aggregated health information can be re-identified.^19–21^ In future, development of approaches such as sharing with family and friends personally via emails or text message could help better promote ItRuns and spread awareness about FHx collection.

Users took about 636 seconds (10.60 minutes) to complete the assessment. This time is lower compared to reported times for FHx collection in-person or using digital health tools (average 15 to 60 minutes).^1,7,8,22^ In a previous study, the previous version of ItRuns took a higher time compared to standard FHx web-based form.^6^ Our initial hypothesis was that ItRuns will require a high completion time resulting in increased abandonment. As ItRuns uses a conversational text message-like interface, users completed certain questions (types of cancer and genetic tests, family member details) multiple times, not aware of these repetitions at the beginning, able to see all information at once, or recollect previously added information without scrolling up the chat history. Also, in the version, users were unable to change any incorrect responses. In contrast to our hypothesis, ItRuns took less time to collect FHx. This could point to the potential benefit of ItRuns in reducing the FHx collection time, while employing an intuitive and friendly interface. Previous research suggests that users perceive chatbots as intuitive, engaging, and friendly for FHx and other health data collection.^6^

Users spent the longest (19.50 seconds) in responding to search list questions. The search lists included cancer types (~ 100 options) and genetic test types (~ 160 options). From the cancer list, ‘Other Cancer’ was the 4th most common option users chose.^23^ As previously reported, the position of the ‘Other Cancer’ option and lack of cancer synonyms (uterine cancer instead of endometrial cancer) could have led to selection of this choice.^23^ Also, if users misspelled a cancer, they might not see the correct option due to the filters. These factors could have led to higher time spent on these subflows. Users also spent about 15 seconds on the question asking for their email address. It is possible that users may have taken time to decide which email address to provide and type it in the text field, which is time consuming. Although, ItRun’s privacy statement ensures users that their personal information will not be shared, an initial hesitation to submit personal email address in a marketing campaign could have resulted in a longer response time. We saw a total of 6.69% abandonment in two text input questions asking for user’s name or family member names, which may likely be due to hesitation to share their own family members’ personal information.

### 4.2 Limitations

The majority of participants were women between the ages 40 and 60 years. The findings may not be representative of the general population. Although, research shows no noticeable age and gender group differences in technology literacy compared to our population.^24^

We aimed to recruit a large sample (over 10,000 individuals) employing a wide cast marketing strategy. This could have resulted in individuals completing the assessment inaccurately or without genuine interest and providing fake data. It is possible that individuals may have completed the assessment in a hurry or taken additional time to complete the assessment at their own convenience. In future research, we aim to recruit a representative clinical population to validate these findings.

The lack of collaboration capabilities for adding personal or family health history within ItRuns could have led to increased times for completing the assessments. Allowing ItRuns users to collaborate with family members and the ability to interact with personal or electronic health records could promote better experience and engagement.

ItRuns collects information in a conversational chat-based approach potentially resulting in higher time to complete the assessment. However, research shows that users prefer the chat-based experience compared to standard, web-based data collection approaches.^6^

The analyses presented in this study were conducted with data extracted from a large-scale software database. The validity and quality of large data sets can vary substantially.^25^ It is particularly important to validate minimum database quality in healthcare contexts.^26^ Our team completed error checks and validations to ensure data integrity.

### 4.3 Future work and implications

This quantitative exploration has future research implications. To the best of our knowledge, we are one of the first teams to conduct a FHx public health campaign to recruit a large-scale sample of over 10,000 individuals and contribute to user experience assessment by analyzing software telemetry data.

Based on this research, we recommend the following considerations to enhance the user experience of end user facing FHx tools and describe how we will improve ItRuns in future.

- *Promote FHx education by embedding education and resources within FHx tools to help users with decision making*. We have already developed features that would allow us to embed tailored education material within Dokbot, such as importance of FHx, hereditary cancer risks, and role of genetic counseling; resources to connect with public health agencies to learn more about these topics, etc. as well as assure users about their information being secure within ItRuns to promote its use without concerns and bridge the potential knowledge gap about FHx collection.
- *Build and maintain trust while collecting FHx by establishing strategies (such as positive reinforcement messages) to alleviate privacy and confidentiality concerns*. Although ItRuns informs users their data will be used for research and not for marketing purposes, we will reassure users when asking about personal and family health history and other identifiable information. We will assure users that ItRuns and Dokbot are compliant to privacy regulations. Patients often trust data sharing recommendations from their providers.^16^ ItRuns can be embedded within patient portals and patient management systems such that providers can directly prescribe it to patients.
- *Empower users and reduce burden by promoting collaboration between family members to gather complete and accurate FHx, reduce FHx collection time, increase adoption, and bridge the knowledge gap*. We aim to allow ItRuns users to collaborate and share assessment with family members and save progress to continue the assessment at a later time without losing previous responses (Save and Return feature). We have implemented features to provide information on progress and time estimates to keep users informed and motivated.

In addition, future research could also address the following:

- This study did not directly study the user’s preferences, but rather via their software usage and actions. Future studies could supplement such analyses with user interviews or focus groups to better understand their experience, perceptions towards ItRuns, and test the hypotheses for assessment abandonment at various stages.
- This study did not aim to assess the quality of data gathered during this study. Future researchers should also consider assessing the accuracy and completeness of the data collected using the chatbot interface.

Lastly, our approach could be readily applied to other healthcare assessments to evaluate user experience and engagement. As a part of this research, we have developed a generalizable program script which could be used to extract and summarize telemetry data of assessments developed using chatbot application- Dokbot.^12^ We aim to continue studying and improving on other custom and standardized Dokbot assessments to enhance patient and provider experience of healthcare data collection.

## 5. Conclusion

This paper quantitatively explores the user experience of ItRunsInMyFamily.com FHx assessment. We used a unique approach by employing software telemetry to assess actual user behaviors related to abandonments and time spent on various ItRuns Steps. The findings provide insights on factors impacting user experience and assessment completions. Key factors include the potential lack of knowledge or interest in FHx, lack of trust in FHx tools, confidentiality concerns about sharing personal and family health information, lack of collaboration with family members, and increased time due to conversational text-based workflow. We discuss considerations for improving FHx collection experience and how our findings will help in the development of the future ItRuns versions for better user experience and engagement.

## Figures and Tables

**Table 1:** Summary of completion rate, departures per step, abandonment, and average time taken for key assessment steps. The time spent on the Goodbye step was not calculated as the users may or may not have closed the browser window after completing the assessment, leading to inaccurate time.

Subflow	Departures Count	Percentage Abandonment	Median Time Spent in Seconds
*Introduction*	3630	32.81	36.00
*Basic Demographics*	74	0.67	7.00
*Proband Cancer History*	532	4.81	124.00
*Proband Gene Testing*	53	0.48	26.50
*Family Cancer History*	1331	12.03	119.00
*Pedigree*	189	1.71	55.00
*Physical Traits*	101	0.91	35.00
*Female Details*	95	0.86	67.00
*Colorectal Cancer Screening*	13	0.12	37.50
*Lifestyle*	63	0.58	53.00
*Thank you and Email*	257	2.32	19.00
*Recommendations/Report*	886	8.01	48.00
*Invite Friends*	2645	23.90	9.00
*Goodbye*	1195	10.80	NA

**Table 2:** Top abandonment by step

Subflow	Step	Abandonment Rate
*Introduction*	“Hi There! :) I’m Dokbot. I will help determine your family’s risk for cancer.”	22.03%
	“I understand and consent”	5.71%
	Thank you. Let’s get started! What’s your first name?	3.80%
*Family Cancer History*	“Alright, I’m now going to ask you a few questions about your family history of cancer.”	5.26%
	“Which cancer did they have?”	2.89%
	“Which relative had cancer?”	2.08%
*Thank you and Email*	“To what email address shall I send your report?”	2.30%
*Recommendations*	“Ok got it. Give me 10–20 seconds to calculate your risk...”	3.84%
*Invite Friends*	“While you are waiting on your report, will you invite your friends and family to use ItRunsInMyFamily so they can learn their family cancer risk as well?”	23.90%
*Goodbye*	“Ok, that’s all for today! Thank you for spending this time with me. If you have any questions or would like to learn more about hereditary cancer, drop by https://itrunsinmyfamily.com.”	10.80%

**Table 3:** Top steps with highest time per step

Subflow	Step	Median Time in Seconds
*Introduction*	Consent	16.00
*Proband Cancer History*	“Which cancer did you have?”	10.00
*Proband Genetic Testing*	“Was the result positive?”	14.50
*Family Cancer History*	“Which relative had cancer?”	10.00
	“Which cancer did he *[she]* have?”	36.00
*Collect Physical Traits*	“Where are your ancestors from?”	20.00
*Thank you and Email*	“To what email address shall I send your report?”	15.00
*Recommendations*	“Ok got it. Give me 10–20 seconds to calculate your risk…”	17.00
	“Alright, based on your family cancer history, it looks like you are at increased risk for cancer. You should discuss these results with your doctor.”	9.00
	“Alright, according to current guidelines and the family history you provided, you do not meet referral criteria for genetic counseling and testing.”	10.00
*Invite Friends*	“While you are waiting on your report, will you invite your friends and family to use ItRunsInMyFamily so they can learn their family cancer risk as well?”	9.00

**Table 4:** Abandonment and time taken per question type

Input type	Total	Percentage Abandonment	Median Time in Seconds
*Button*	27	68.67	8.38
*Input- text*	2	6.69	7.00
*Radio input*	3	5.27	7.67
*No input*	1	4.84	17.00
*Input- number*	17	4.09	6.06
*Search List*	4	4.05	19.50
*Input- email*	1	2.30	15.00
*Button- Yes* / *No*	24	1.74	5.32
*Number input with +/−*	9	1.07	5.67
*Checklist*	5	0.98	11.20
*Button- Yes* / *No* / *IDK*	1	0.28	14.50

## References

[1] Welch BM, Wiley K, Pflieger L, Review and Comparison of Electronic Patient-Facing Family Health History Tools. J Genet Couns 2018; 27: 381–391. 29512060 10.1007/s10897-018-0235-7 PMC5861014

[2] Miroševič Š, Krajc K, Klemenc-Ketiš Z, Mapping Users’ Experience of a Family History and Genetic Risk Algorithm Tool in Primary Care. Public Health Genomics 2021; 1–10. 10.1159/000518086 34515220

[3] Welch BM, O’Connell NS, Qanungo S, Collecting Family Health History using an Online Social Network: a Nationwide Survey among Potential Users. AMIA Annu Symp Proc 2015; 2015: 1316–1325. 26958272 PMC4765590

[4] Cohn WF, Ropka ME, Pelletier SL, Health Heritage© a web-based tool for the collection and assessment of family health history: initial user experience and analytic validity. Public Health Genomics 2010; 13: 477–491. 20424421 10.1159/000294415

[5] Jovanovic M, Baez M, Casati F. Chatbots as conversational healthcare services. IEEE Internet Comput 2020; 1–1.

[6] Ponathil A, Ozkan F, Welch B, Family health history collected by virtual conversational agents: An empirical study to investigate the efficacy of this approach. J Genet Couns 2020; 29: 1081–1092. 32125052 10.1002/jgc4.1239

[7] Arar N, Seo J, Abboud HE, Veterans’ experience in using the online Surgeon General’s family health history tool. Per Med 2011; 8: 523–532. 22076122 10.2217/pme.11.53 PMC3210025

[8] Bajracharya AS, Crotty BH, Kowoloff HB, Patient experience with family history tool: analysis of patients’ experience sharing their family health history through patient-computer dialogue in a patient portal. J Am Med Inform Assoc 2019; 26: 603–609. 30946464 10.1093/jamia/ocz008 PMC7647187

[9] Wang C, Paasche-Orlow MK, Bowen DJ, Utility of a virtual counselor (VICKY) to collect family health histories among vulnerable patient populations: A randomized controlled trial. Patient Educ Couns 2021; 104: 979–988. 33750594 10.1016/j.pec.2021.02.034 PMC8113103

[10] Dugar V. Telemetry in Software. justDevTalk, https://medium.com/justdevtalk/telemetry-in-software-7e2766a58cc0 (2019, accessed 30 September 2021).

[11] Family health history, https://www.cdc.gov/genomics/famhistory/index.htm (2021, accessed 25 October 2021).

[12] Dokbot: A better way to collect data from patients, https://dokbot.io/ (accessed 1 October 2021).

[13] Zubko A. Why Do Ashkenazi Jews Have a Higher Risk of Breast Cancer?, https://www.maurerfoundation.org/why-do-ashkenazi-jews-have-a-higher-risk-of-breast-cancer/ (2019, accessed 15 October 2021).

[14] Ritchie JB, Frey LJ, Lamy J-B, Automated Clinical Practice Guideline Recommendations for Hereditary Cancer Risk Using Chatbots and Ontologies: System Description. JMIR Cancer 2022; 8: e29289. 35099392 10.2196/29289 PMC8845001

[15] Linder C. Clemson University. MS Industrial Engineering, Clemson University, https://search.proquest.com/openview/135121eed33b0f3434aae1ad9a6d4636/1?pq-origsite=gscholar&cbl=18750&diss=y (2020).

[16] Soni H, Grando A, Murcko A, State of the art and a mixed-method personalized approach to assess patient perceptions on medical record sharing and sensitivity. J Biomed Inform 2020; 101: 103338. 31726102 10.1016/j.jbi.2019.103338 PMC6952579

[17] Welch BM, Dere W, Schiffman JD. Family health history: the case for better tools. JAMA 2015; 313: 1711–1712. 25868012 10.1001/jama.2015.2417

[18] Nurgalieva L, Cajander Å, Moll J, ‘I do not share it with others. No, it’s for me, it’s my care’: On sharing of patient accessible electronic health records. Health Informatics J 2020; 26: 2554–2567. 32264734 10.1177/1460458220912559

[19] Bender JL, Cyr AB, Arbuckle L, Ethics and Privacy Implications of Using the Internet and Social Media to Recruit Participants for Health Research: A Privacy-by-Design Framework for Online Recruitment. J Med Internet Res 2017; 19: e104. 28385682 10.2196/jmir.7029 PMC5399223

[20] Na L, Yang C, Lo C-C, Feasibility of Reidentifying Individuals in Large National Physical Activity Data Sets From Which Protected Health Information Has Been Removed With Use of Machine Learning. JAMA Netw Open 2018; 1: e186040. 30646312 10.1001/jamanetworkopen.2018.6040 PMC6324329

[21] Braunack-Mayer A, Fabrianesi B, Street J, Sharing Government Health Data With the Private Sector: Community Attitudes Survey. J Med Internet Res 2021; 23: e24200. 34596573 10.2196/24200 PMC8520136

[22] Cerda Diez M E Cortés D, Trevino-Talbot M, Designing and Evaluating a Digital Family Health History Tool for Spanish Speakers. Int J Environ Res Public Health; 16. Epub ahead of print 7 December 2019. DOI: 10.3390/ijerph16244979. PMC6950582 31817849

[23] Welch BM, Allen CG, Ritchie JB, Using a Chatbot to Assess Hereditary Cancer Risk. JCO Clin Cancer Inform 2020; 4: 787–793. 32897737 10.1200/CCI.20.00014 PMC7529541

[24] Mamedova S, Pawlowski E. A Description of U.S.Adults Who Are Not Digitally Literate. US Department of Education, May 2018.

[25] Nagle T, Redman T, Sammon D. Assessing data quality: A managerial call to action. Bus Horiz 2020; 63: 325–337.

[26] Kilkenny MF, Robinson KM. Data quality: ‘Garbage in - garbage out’. Health Inf Manag 2018; 47: 103–105. 29719995 10.1177/1833358318774357

